# Supplementary material for: The novel nematicide chiricanine A suppresses Bursaphelenchus xylophilus pathogenicity in Pinus massoniana by inhibiting Aspergillus and its secondary metabolite, sterigmatocystin
Source: Front Plant Sci. 2023 Nov 8;14:1257744. doi: 10.3389/fpls.2023.1257744 (PMC10663349; doi:10.3389/fpls.2023.1257744)
Supplement: Supplementary file 1 [file DataSheet_1.zip › Supplementary-Figures.docx]

**
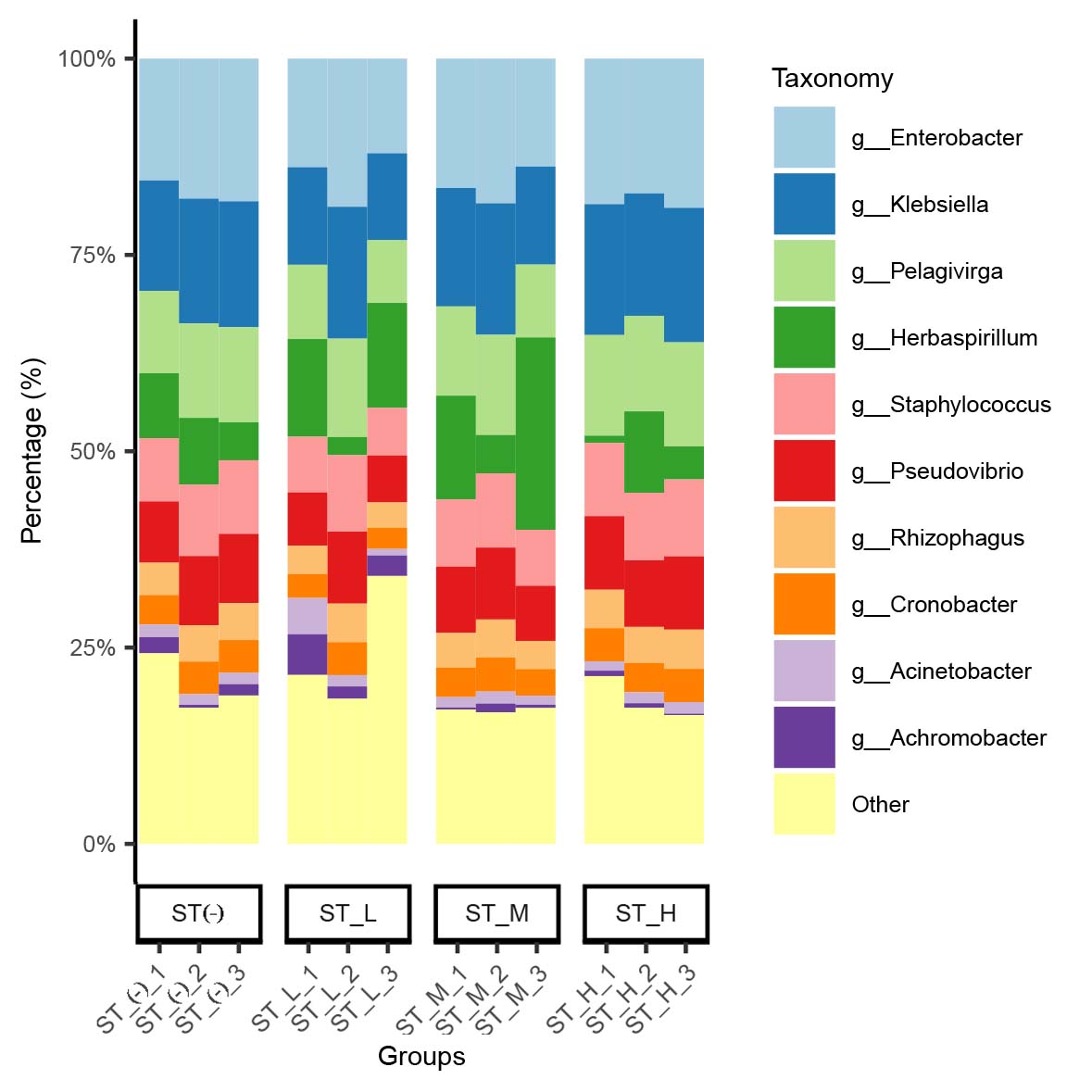
**

**Supplementary Figure S1 | Correlations between ST and their correlated microbes in *Pinus massoniana*.**

**
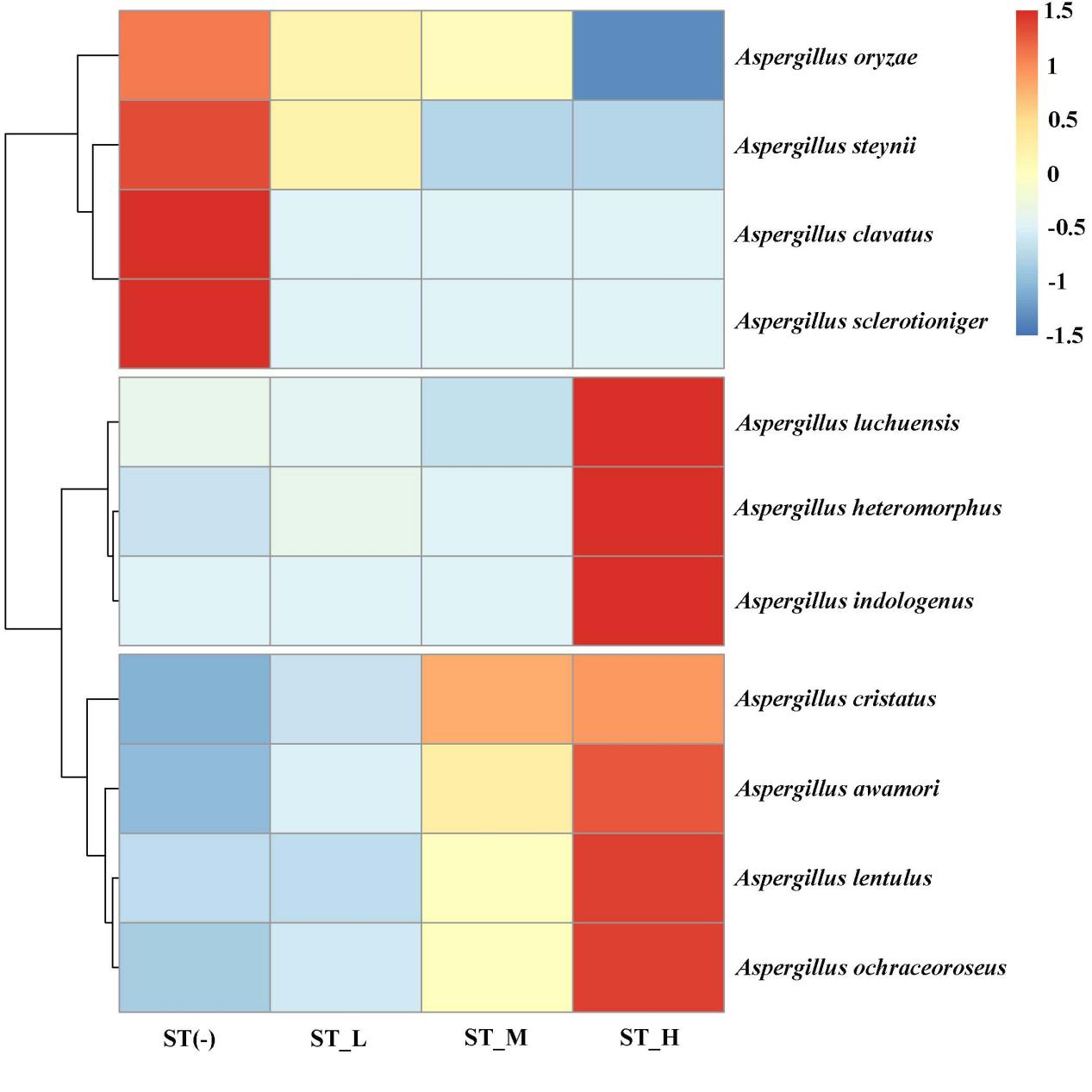
**

**Supplementary Figure S2 | Correlations between ST and their correlated microbes of *Aspergillus* genus of fungi in *Pinus massoniana*.**
